# Liver X receptor activation reduces gastric cancer cell proliferation by suppressing Wnt signalling via LXRβ relocalization

**DOI:** 10.1111/jcmm.13974

**Published:** 2018-10-19

**Authors:** Qiang Wang, Fan Feng, Jiayou Wang, Meijia Ren, Zhonggang Shi, Xiang Mao, Heng Zhang, Xiaoli Ju

**Affiliations:** ^1^ Institute of Life Sciences Jiangsu University Zhenjiang China; ^2^ The Fourth Affiliated Hospital of Jiangsu University Zhenjiang China; ^3^ School of Medicine Jiangsu University Zhenjiang China; ^4^ Department of General Surgery Nanjing Lishui District People's Hospital Zhongda Hospital Lishui Branch Southeast University Nanjing China

**Keywords:** cell proliferation, gastric cancer, LXRβ, translocation, Wnt signalling

## Abstract

Liver X receptors (LXRs) are involved in various diseases associated with lipid disorders, and in regulating cancer cell proliferation. However, the underlying molecular mechanisms, especially those in gastric cancer (GC) remain to be clarified. In this study, immunohistochemistry analysis revealed that LXRβ was mainly expressed in GC tissue, with less expression in adjacent normal tissues. The LXRβ agonist T0901317 efficiently suppressed the proliferation and colony formation of various GC cell lines. We further showed that LXRβ translocated from the cytoplasm to the nucleus when activated by T0901317. LXRβ nuclear localization suppressed the activation of Wnt signalling and decreased the expression of target genes such as MYC, BMP4, and MMP7 through binding to their promoters. Moreover, we demonstrated that the LXR agonist efficiently suppressed GC tumour growth in a nude mouse xenograft model. Taken together, these results revealed that LXRβ agonist inhibited GC cells proliferation by suppressing Wnt signalling via LXRβ relocalization. The results strongly suggest that LXRβ could be a promising target in GC therapy.

## 1 INTRODUCTION

Liver X receptors (LXRs) are important members of the nuclear receptor (NR) superfamily that are involved in a number of diseases, including lipid disorders, cancer, and neurodegenerative diseases.^1^ Two isoforms of LXR, LXRα (NR1H3), and LXRβ (NR1H2), were initially identified as orphan receptors, and each isoform has distinct tissue expression and function.^2^, ^3^ LXRβ is ubiquitously expressed in all tissues, and there is accumulating evidence to support that LXRs are involved in a variety of cancers by different mechanisms and are potential targets in cancer therapeutics.^2^, ^4^

One of the main mechanisms by which LXR agonist inhibits tumour growth is through inhibition of cell proliferation and induction of cell death.^3^ In pancreatic ductal adenocarcinoma (PDAC), LXR agonist treatments inhibit cell proliferation, cell‐cycle progression, and colony formation, regulating multiple gene networks involved in cell cycle arrest and growth factor signalling.^5^ LXR agonists also inhibit cell proliferation and cell cycle arrest in breast cancer cells by regulating hepatic expression of the oestrogen deactivation enzyme.^6^ It was also reported that LXR activation leads to cell death through pyroptosis in colon cancer.^7^, ^8^, ^9^ Conversely, LXR agonist induces apoptosis in LNCaP cells and reduces the growth of xenograft LNCaP tumours in nude mice.^10^ However, whether LXR agonists have an effect on gastric cancer (GC) growth needs to be clarified.

The subcellular localization of LXR is controversial in different cancer cells. It was previously reported that unliganded LXRα mainly localizes the nucleus in a nuclear localization signal‐dependent manner, whereas unliganded LXRβ is partially exported from the nucleus.^11^, ^12^ In contrast, LXRβ shows predominant cytoplasmic localization in colon cancer cells but not in normal colon mucosa cells.^8^, ^9^ Both nuclear and cytoplasmic localization was observed in PDAC samples.^5^

In this study, we investigated whether LXR agonists inhibit the growth of GC cells and the underlying mechanism of inhibition. We found that LXR agonists inhibit the proliferation of various GC cell lines. Furthermore, LXRβ exhibits different intracellular localization when stimulated with an LXRβ agonist. The nuclear localization of LXRβ after agonist stimulation correlates with the suppression of Wnt signalling. Finally, the in vivo experiment demonstrated that the LXR agonist suppresses tumour growth in a nude mouse model.

## 2 EXPERIMENTAL PROCEDURES

### 2.1 Cell culture and animal studies

Human AGS, AZ521, SGC, BGC, and MGC cells were obtained from American Type Culture Collection (ATCC, Manassas, VA, USA). All cell lines were cultured in RPMI 1640 or DMEM (Gibco, Carlsbad, CA, USA) supplemented with 10% foetal bovine serum (Gibco, Carlsbad, CA, USA) and pen/strep (100 μg/mL). T0901317 was purchased from Cayman Chemical Company (Ann Arbor, MI, USA).

The animal experiments were approved by the Ethics Committee of Jiangsu University. For xenograft experiments, 3 × 10^6^ SGC cells were subcutaneously injected into 6 to 8‐week‐old BALB/c athymic nude mice. Two days after cell injection, the mice were intraperitoneally treated with T0901317 (50 μg/g mouse) every 3 days. Tumour volume was measured every 5 days with a caliper.

### 2.2 Immunostaining and immunocytochemistry

The cells were seeded on coverslip glasses, fixed in formaldehyde (4% in PBS) and permeabilized with 0.1% Triton X‐100 in PBS. Subsequently, the cells were incubated with LXRβ antibodies (Cell Signaling, Boston, MA, USA) for 30 minutes, after which Alexa Fluor 488 goat anti‐rabbit IgG (Proteintech, Wuhan, China) was added, and the cells were incubated for 30 minutes. Stained cells were observed under a fluorescence microscope. The relative percent fluorescence was calculated by ImageJ software. Immunocytochemistry were performed as previously described.^13^ Briefly, samples were deparaffinized, rehydrated, and incubated in Antigen Retrieval Citra Solution. After being blocked in 5% BSA, the sections were incubated with LXRβ antibodies (1:200 dilution) (Cell Signaling) overnight at 4°C. Then, anti‐rabbit IgG SABC and DAB detection kits were used to detect the signals.

The expression levels of LXRβ in tumour cells were scored as the intensity of staining and the percentage of positive‐stained cells. LXRβ expression was graded into three groups: 0 (no positive cells indicates no staining), 1 (cells presented yellowish indicate weak staining), 2 (light‐brown and dark‐brown staining indicate intense staining). The percentage of LXRβ expression was determined based on 10 random areas (HPF 400× magnification) in each section, and the values were averaged for statistical analysis; 1000 tumour cells were counted for LXRβ expression analysis. Cases in group 2 with ≥20% LXRβ in tumour cells were considered to have high LXRβ expression.

### 2.3 Western blot analysis

Cells were lysed in RIPA lysis buffer according to the manufacturer's protocol ((BOSTER, Wuhan, China). Immunoblot analyses were conducted as previously described.^5^ LXRβ (1:1000 dilution) and c‐Myc (1:1000 dilution) (Cell Signaling), BMP4 and MMP7 (1:500 dilution) (BBI, Shanghai, China) and GAPDH (1:2000 dilution) (Proteintech) antibodies were used as primary antibodies. Then, goat anti‐rabbit IgG (Proteintech) was used as the secondary antibody. An enhanced chemiluminescence detection system was used to detect the signals.

### 2.4 Proliferation and colony formation assay

For analysis of cell proliferation, cells were cultured in 96‐well plates overnight and, then treated with LXR agonists. After treatment, WST‐1 reagents were added to the cell culture medium, and the cells were incubated for 2 hours. The absorption at 450 nm was determined using a BioTek microplate reader.

For BrdU assays, cells grown in 6‐well plates were incubated with 10 μmol L^−1^ T0901317 for 48 hours. Then, the cells were incubated with 1 mg/mL BrdU for 48 hours. Cells were fixed, permeabilized, and blocking with 3% BSA for 1 hour. Next, the cells were incubated with anti‐BrdU antibody at 4°C overnight. Then, FITC‐labelled anti‐rat IgG was added for 1 hour. Cells were observed under a fluorescence microscope and counted.

For colony formation assays, 2 × 10^2^ cells were seeded in 10‐cm plates and treated with the LXR agonist for 10 days. Then, the cells were washed, fixed with 4% formaldehyde, stained with crystal violet (Sigma‐Aldrich, St. Louis, MO, USA) and counted under a microscope.

### 2.5 Chromatin immunoprecipitation assay

ChIP assays were performed using the ChIP Assay Kit (Millipore, Billerica, MA, USA) according to the manufacturer's protocol. Briefly, chromatin was cross‐linked at RT for 10 minutes with 1% formaldehyde in medium; 200 μL lysis buffer was added, and the supernatant was suspended in elution buffer. Immunoprecipitation was performed for 8 hours at 4°C with anti‐LXRβ antibodies (1:50 dilution) (Cell Signaling) or normal IgG as a negative control. Protein A agarose was added for 1 hour at 4°C to collect the protein‐antibody complexes. After washing, immunocomplexes were eluted in elution buffer. Finally, the free precipitated DNA was purified with a PCR Purification Kit (BBI). PCR was performed using ExTaq (Takara, Kyoto, Japan) following the manufacturer's protocol. The primer sequences were 5′‐TTGCTGGGTTATTTTAATCAT‐3′ and 5′‐ACTGTTTGACAAACCGCATCC‐3′ for the c‐Myc promoter and 5′‐GATACCTATGAGAGCAGTCA‐3′ and 5′‐CTGCTAGTGACTGCAGAAAT‐3′ for the MMP7 promoter.

### 2.6 Wound healing and transwell invasion assay

AGS cells were plated in 6‐well plates. When cell confluence reached approximately 80%, the cell monolayers were wounded with a 20 μL sterile pipette tip and washed with PBS. The cells were then cultured in serum‐free medium containing the indicated concentration of T0901317. The wounded cell monolayers were cultured for another 48 hours and visualized by microscopy to assess cell migration ability. The experiments were performed in triplicate.

Cell invasion was determined using Matrigel‐coated (BD Biosciences, San Jose, CA, USA) transwell membranes chambers (diameter 6.5 mm, pore size 8 μm) (Corning Costar, Cambridge, MA, USA). Cells were cultured in serum‐free medium for 24 h and then reseeded into the upper chamber of the transwell insert, with or without T0901317. Medium with 10% FBS was added to the lower chamber, and the samples were incubated at 37°C for 24 hours. Noninvading cells in the upper chamber were removed with a cotton swab, and invasive cells were fixed with 4% paraformaldehyde and stained with 0.1% crystal violet.

### 2.7 Statistical analysis

All group data are shown as the mean ± SD Error bars indicate the range of values from duplicate experiments. Differences between groups were analysed by Student's t‐test using GraphPad Prism5 software. *P* < 0.05 was regarded as a statistically significant difference. All experiments were repeated at least in duplicate with triplicate technical replicates.

## 3 RESULTS

### 3.1 LXRβ is elevated in gastric cancer tissues

**Figure 1:**
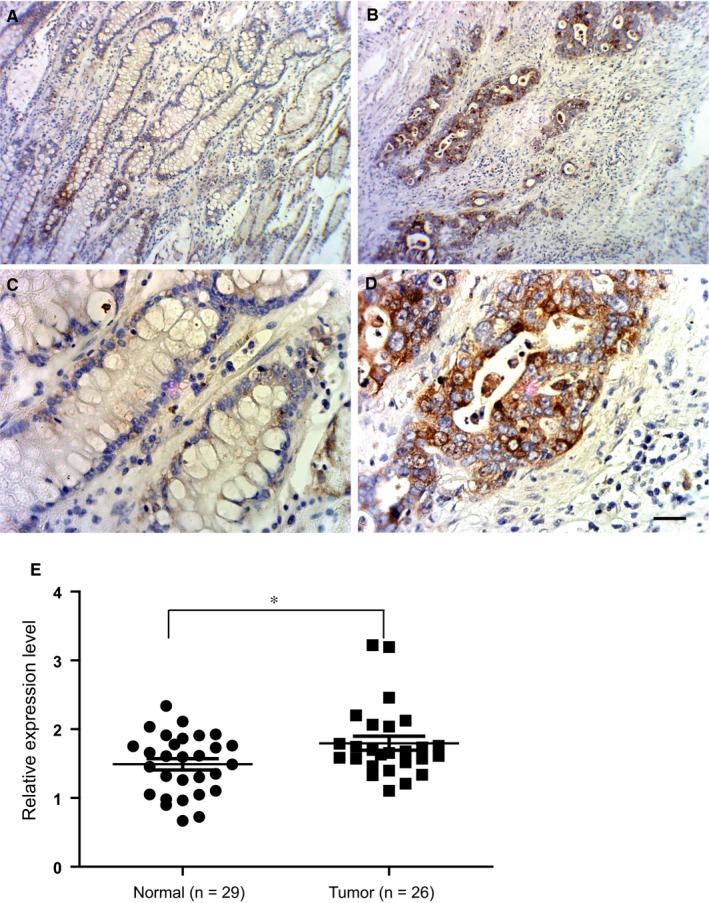
LXRβ expression level in GC tissues and adjacent normal tissues. (A–D) Immunohistochemical staining of LXRβ in GC tissues (A and C) and adjacent normal tissues (B and D). (original magnification: A and B, ×100, C and D, ×400). (E) LXRβ mRNA expression level in GC tissues (n = 29) and adjacent normal tissues (n = 26) from Oncomine. *P < 0.05 with the unpaired t‐test

We first examined LXRβ expression in GC tissues and adjacent normal tissues in clinical samples. Immunohistochemical staining revealed that LXRβ was mainly expressed in GC tissues (Figure 1B and D), with less expression in adjacent normal tissues (Figure 1A and C). LXRβ were mainly expressed in the cytoplasm in GC tissues and adjacent normal tissues. (Figure 1). Next, we analysed LXRβ expression in 55 paired tumour and normal tissues from the Oncomine dataset (https://www.oncomine.org). As shown in Figure 1E, the LXRβ mRNA expression level was higher in GC tissues than in normal tissues. Similar results for LXRβ expression were also observed at the protein level by immunohistochemical staining (Figure 1A–D). An analysis of the clinical features of the GC samples revealed that LXRβ staining was higher in GC tissue than in adjacent normal tissue in 66.7% (36/54) of the GC patients. Statistical significance of LXRβ expression was also observed during disease stage I or II (Table 1). Taken together, these data suggest that LXRβ expression is increased in GC tissues.

**Table 1:** The correlation of LXR‐β expression to clinical features of gastric cancer

Characteristic	n	High LXR‐β (n = 36)	Low LXR‐β (n = 18)	*P*‐value
Gender				
Male	42	29 (69%)	13 (31%)	0.806
Female	12	7 (58%)	5 (42%)	
Age (years)				
>60	29	18 (62%)	11 (38%)	0.912
≤60	25	15 (60%)	10 (40%)	
Tumour differentiation				
Poor or moderate	41	24 (59%)	17 (41%)	0.750
Well	13	8 (62%)	5 (38%)	
Disease stage				
I or II	19	9 (47%)	10 (53%)	**0.01**
III or IV	35	24 (69%)	11 (31%)	
Primary tumour size (cm)				
<5	21	12 (57%)	9 (43%)	0.237
≥5	33	20 (61%)	13 (39%)	
Lymph node				
Negative	18	10 (56%)	8 (44%)	0.562
Positive	36	22 (61%)	14 (39%)	

*P*‐values <0.05 are indicated in bold.

### 3.2 LXRβ is mainly expressed in the cytoplasm in human gastric cancer cell lines

**Figure 2:**
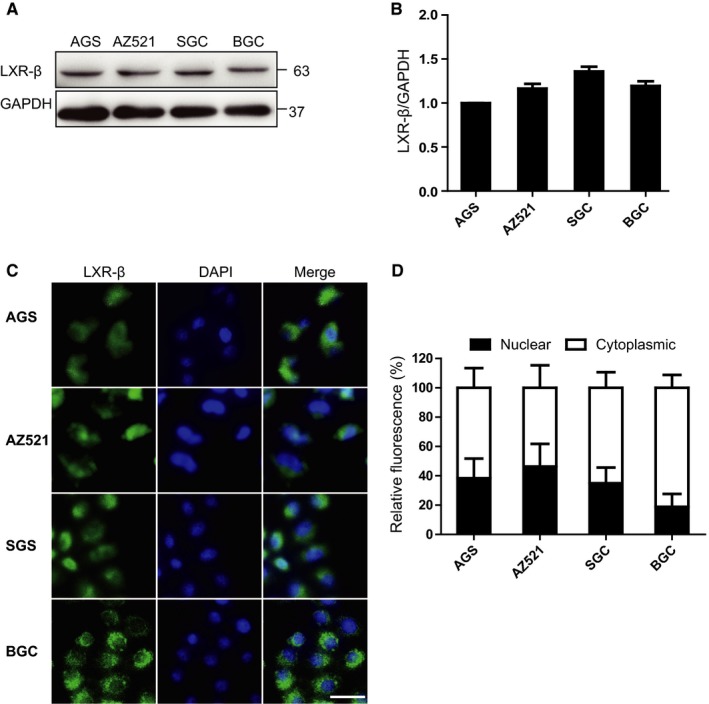
LXRβ expression and localization in GC cell lines. (A) Western blot analysis of LXRβ expression in the AGS, AZ521, SGC, and BGC cell lines. GAPDH was used as the internal control. (B) Relative LXRβ expression levels in (A) were quantified in the histograms by ImageJ software. (C and D) The indicated cells were stained with an LXRβ antibody and the nuclear marker DAPI and observed under a fluorescence microscope. Scale bar, 20 μm. The relative LXRβ fluorescence in the nucleus and cytoplasm of indicated cells was quantified by ImageJ software

We then utilized Western blot analysis to evaluate LXRβ expression levels in different human GC cell lines. As expected, LXRβ protein was expressed in all the examined human GC cell lines, including AGS, AZ521, SGC, BGC, and MGC cells (Figure 2A and B). The subcellular localization of LXRβ in these cell lines was examined by immunofluorescence staining. LXRβ was detected in both the nucleus and cytoplasm (Figure 2C and D), but its expression was much higher in the cytoplasm than in the nucleus, especially in the SGC and BGC cell lines (approximately 60%‐80% cytoplasmic LXRβ) (Figure 2D).

### 3.3 LXR agonist reduces the growth of gastric cancer cells

**Figure 3:**
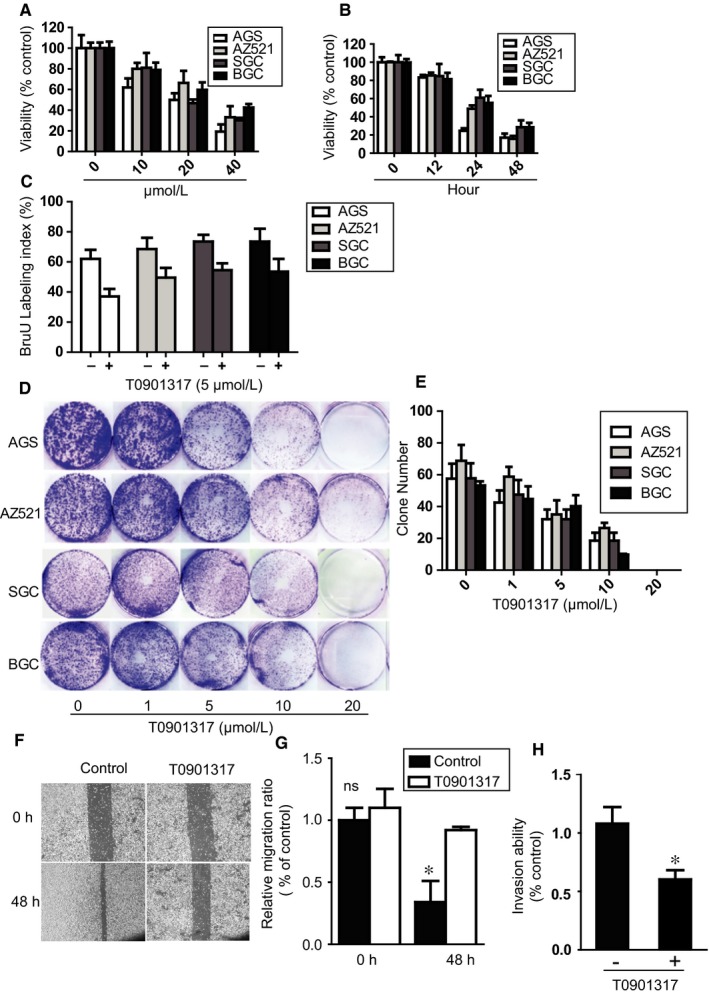
The LXR agonist blocks GC cell proliferation and colony formation. (A and B) The indicated cells were stimulated with 0‐40 μmol L^−1^ T0901317 for 24 h (A) or with 40 μmol L^−1^ T0901317 for 0 to 48 h. Cell viability was analysed by WST‐1 assay. (C) Cells were stimulated with 5 μmol L^−1^ T0901317 for 72 h, and the BrdU labelling index was measured. (D and E) The indicated cells were treated with 0‐20 μmol L^−1^ T0901317 for 10 d. Colony formation ability was observed under a microscope (D), and colony numbers were quantified (E). (F and G) AGS cells were stimulated with or without 5 μmol L^−1^ T0901317 for 48 h. The wound healing assay was used to examine migration ability under a microscope (F), and the relative migration ratio was calculated (G). (H) AGS cells were stimulated with or without 5 μmol L^−1^ T0901317 for 24 h, and invasion ability was quantified. Data are shown as the mean ± standard deviation (SD). *P < 0.05 (two‐tailed Student's t‐test)

Next, we tested whether the synthetic LXR agonist T0901317 affects the viability and proliferation of human GC cell lines. AGS, AZ521, SGC, and BGC cells were treated with different doses of T0901317, and cell viability was determined by WST‐1 assay. The LXR agonist T0901317 significantly reduced the viability of these cell lines at 20‐40 μmol L^−1^ (Figure 3A). All the cell lines showed significantly decreased viability from 24 to 48 hours after treatment (Figure 3B). We then examined whether inhibiting cell proliferation contributed to the reduction in GC cell numbers. The BrdU assay showed that cell proliferation was significantly decreased after treatment with 5 μmol L^−1^ T0901317 for 72 hours (Figure 3C). We then examined the effect of T0901317 on the proliferation and colony formation of GC cell lines in colony formation assays. As shown in Figure 3D and E, treatment of AGS, AZ521, SGC, and BGC cells with 1‐20 μmol L^−1^ T0901317 for 72 hours significantly inhibited colony formation compared with control treatment. Because of statistical significance between LXRβ expression and disease staging (*P* = 0.010), we also examined the impact of T0901317 on cell migratory and invasive potentials by wound healing and transwell assays. The results showed that T0901317 significantly inhibited cell migration when compared with the control (Figure 3F and G). T0901317 also the inhibited the invasion capacity of AGS cells (Figure 3H). Taken together, these data suggest that LXR agonists reduce GC cell growth by inhibiting proliferation.

### 3.4 LXR agonist induces LXRβ subcellular relocalization in gastric cancer cells

**Figure 4:**
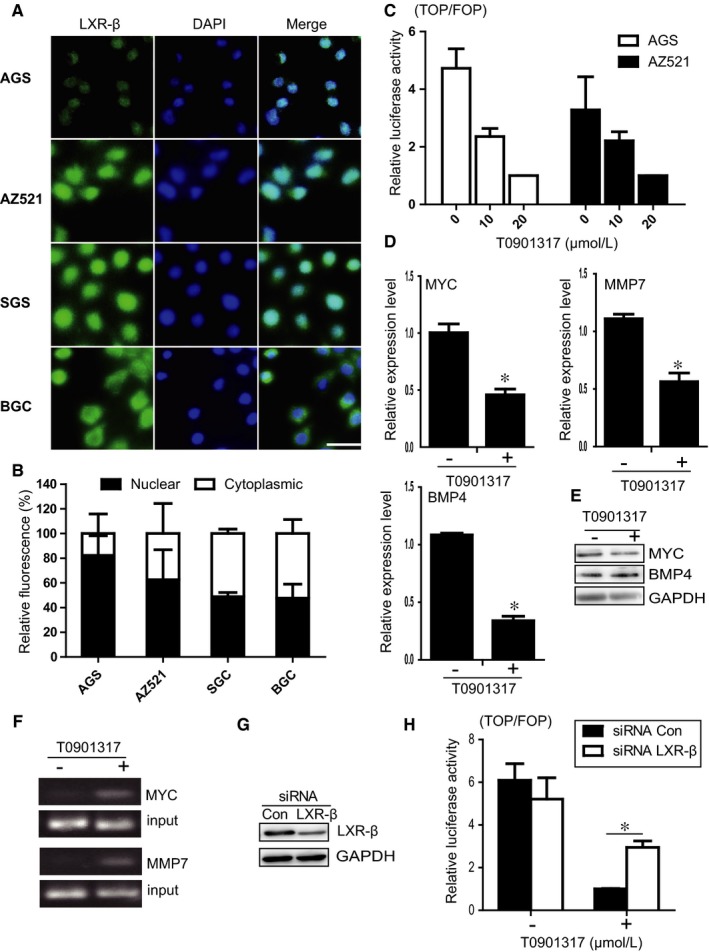
T0901317 induces LXRβ nuclear relocalization and suppresses Wnt signalling. (A) The indicated cells were stimulated with 5 μmol L^−1^ T0901317 for 6 h and then stained with an LXRβ antibody and the nuclear marker DAPI. Scale bar, 20 μm. (B) The relative LXRβ fluorescence in the nucleus and cytoplasm of the indicated cells was quantified by ImageJ software. (C) Relative luciferase activity of TOPflash and FOPflash in AGS and AZ521 cells stimulated with 0‐20 μmol L^−1^ T0901317. (D and E) Relative expression levels of MYC, MMP7, and BMP4 in AGS cells treated with or without T0901317, as determined by RT‐PCR (D) or Western blotting (E). (F) ChIP analysis of the MYC and BMP4 promoter regions using AGS cell lysates immunoprecipitated (IP) with the anti‐LXRβ antibody. (G) AGS cells were transfected with siRNAs against LXRβ for 48 h. The expression of LXRβ was assessed by Western blotting. (H) AGS cells were transfected with siRNAs against LXRβ for 48 h. Then cells were treated with 20 μmol L^−1^ T0901317. Relative luciferase activity of TOPflash and FOPflash were measured. Data are shown as the mean ± standard deviation (SD). *P < 0.05 (two‐tailed Student's t‐test)

Under unstimulated activation, LXRβ was mainly localized in the cytoplasm of GC cells (Figure 2C and D). When AGS, AZ521, SGC, and MGC cells were treated with the LXR agonist T0901317 for 12 hours, LXRβ relocalized to the nucleus (Figure 4A). As shown in Figure 4B, approximately 80% and 60% AGS and AZ521 cells, respectively, showed nuclear localization after T0901317 treatment. Nuclear relocalization of LXRβ also significantly increased in SGC and BGC cells after T0901317 treatment (Figures 2D and 4B).

### 3.5 LXR agonist suppresses Wnt signalling in gastric cancer cells

We then examined the mechanisms by which the LXR agonist inhibits proliferation of GC cells. As LXRβ relocalized to the nucleus after activation, we investigated whether LXRβ is involved in activating the Wnt signalling pathway. TOP/FOP reporter assays were conducted to examined Wnt signalling activation in a β‐catenin‐dependent manner.^14^ As AGS and AZ521 cells showed greater nuclear localization of LXRβ after treatment, we examined Wnt signalling activation in these two cell lines. As shown in Figure 4C, the luciferase reporter analysis revealed that Wnt signalling activity was significantly inhibited in both AGS and AZ521 cells pretreated with the LXR agonist T0901317. Notably, Wnt activation levels decreased in accordance with the concentration of T0901317 (Figure 4C). Next, we assessed whether the LXR agonist affects expression of Wnt signalling target genes, such as MYC, BMP4, and MMP7,^15^ in GC cells. MYC, BMP4, and MMP7 expression was decreased in AGS cells treated with T0901317 (Figure 4D). The protein expression level of MYC was also slightly increased in AGS cells after treatment (Figure 4E). We then performed chromatin immunoprecipitation (ChIP) assays to determine whether LXRβ is recruited to the promoters of Wnt target genes. As shown in Figure 4F, LXRβ was recruited to the promoters of the MYC and MMP7 genes in the presence of T0901317. To further confirm that LXR agonist suppresses Wnt signalling in gastric cancer cells by the inhibition of LXRβ, LXRβ expression were suppressed by siRNA (4G). As shown in Figure 4 H, luciferase reporter analysis revealed that Wnt signalling activity was increased when LXRβ expression were suppressed in AGS cells. Taken together, these data suggest that the LXR agonist inhibits GC cell proliferation by suppressing the activation of Wnt signalling.

### 3.6 LXR agonist suppresses tumour growth in nude mice

**Figure 5:**
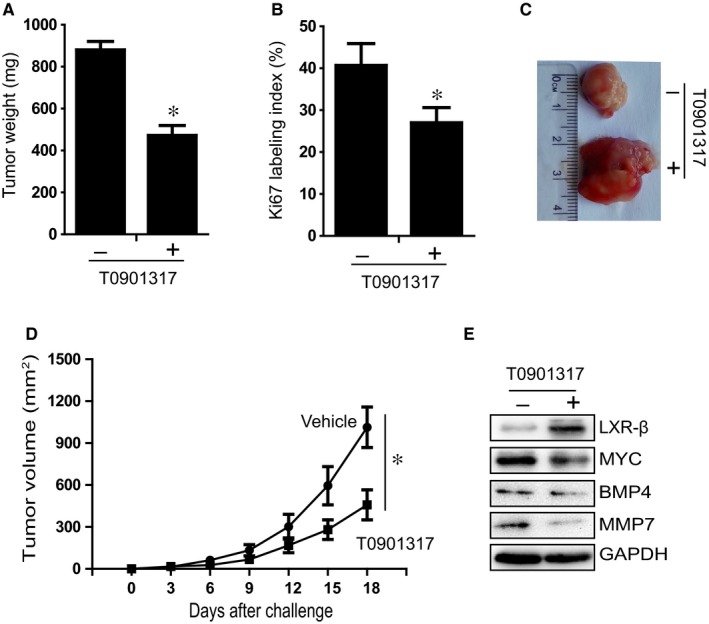
The LXR agonist decreases GC cell xenograft tumour growth in nude mice. (A) BALB/c athymic nude mice were subcutaneously injected with SGC cells, and intraperitoneally treated with T0901317 every 3 d beginning 2 d after cell inoculation. Tumour weight was measured on day 15. (B) The Ki67 labelling index was used to evaluate cell proliferation in SGC xenografts. (C) The photographs of excised tumours at 15 d postinoculation. (D) Tumour volume was measured every 3‐5 d using a caliper. (E) Relative expression levels of LXRβ, MYC, MMP7, and BMP4 in nude mice xenograft tissues treated with or without T0901317, as determined by Western blotting. Data are shown as the mean ± standard deviation (SD). *P < 0.05 (two‐tailed Student's t‐test)

To determine whether the LXR agonist suppresses tumour growth in vivo, we ascertained the effect of T0901317 on the growth of xenografts in nude mice. As shown in Figure 5A, tumour weight in nude mice was significantly reduced after treatment with T0901317 for 15 days. Moreover, Ki67 expression in tumour cells was significantly decreased in mice treated with T0901317 for 15 days (Figure 5B). Furthermore, the tumour volume of T0901317‐treated mice was significantly lower than that of control mice (Figure 5C and 5D). We also examined protein expression of LXRβ, MYC, MMP7, and BMP4 in nude mice xenograft tissues. As shown in Figure 5E, LXRβ expression level was increased and MYC, MMP7, and BMP4 slightly decreased after treatment with T0901317. These results were consistent with the in vitro experimental results.

## 4 DISCUSSION

In this study, we demonstrated that LXRβ was strongly expressed in GC tissues from clinical samples at both the mRNA and protein levels. Interestingly, LXRβ expression was much weaker in adjacent normal tissue than in GC tissue (Figure 1). These findings indicate that LXRβ is abnormally expressed in GC tissues from patients, which is consistent with the findings of a previous report.^5^ In pancreatic cancer, LXRβ can be detected in pancreatic adenoma clinical samples and in all pancreatic cell lines.^5^ LXRβ expression during disease stage I or II was also statistical significance (Table 1), suggesting that LXRβ may be a potential target during the early stage of gastric cancer. Consistent with our observations in clinical samples, we also determined that LXRβ was expressed in various GC cell lines. Colon cancer patient tissues, but not normal colon mucosa cells, were sensitive to treatment with LXR agonists,^9^, ^16^ suggesting that LXRβ could be a promising target in cancer therapy. We also found that subcutaneous injection of an LXR agonist into nude mice suppressed human GC xenograft growth.

The localization of LXRβ in cells of different cancer types is controversial. It was reported that unliganded LXRβ is mainly localized in the nucleus.^11^ However, in colon cancer, unliganded LXRβ shows a predominant cytoplasmic localization.^9^ Here, we revealed that LXRβ had different subcellular localization patterns during activation. Unliganded LXRβ was mainly expressed in the cytoplasm, whereas liganded LXRβ showed nuclear localization after activation in human GC cells (Figures 2 and 4). The different subcellular localization of LXRβ correlated with the function of LXRβ in inhibition GC cell growth. There results suggest that the differential localization of LXRβ in cancer cells is critical for inducing cell death or inhibiting cellular growth.

The Wnt signalling pathway plays a critical role in GC initiation and progression. Several key components of the Wnt pathway have been reported to be overexpressed in gastric carcinogenesis.^17^ In this study, we demonstrated that LXRβ translocated into the nucleus and suppressed Wnt signalling through recruitment to the promoters of Wnt target genes. The expression levels of Wnt target genes, including MYC, BMP4, and MMP7, were significantly suppressed after treatment with T0901317. MYC is required for the activation of the majority of Wnt target genes in colon carcinogenesis.^18^ In agreement with our result, LXRβ activation controls MYC gene expression in colon cancer and prostate cancer.^7^, ^19^, ^20^ BMP4 expression level was also suppressed in colon cancer and MEFs after stimulation with an LXR agonist.^21^, ^22^ This suppression is associated with activation of Wnt signalling.^23^ Although a previous report showed that LXRβ interacts with β‐catenin in colon cancer cells,^20^ we did not detect direct interaction of LXRβ and β‐catenin in gastric cell lines. It is possible that LXRβ interacts with other proteins in gastric cell lines to suppress Wnt signalling activation. Further work is needed to clarify this point. Taken together, these results suggest that LXRs are involved in the Wnt pathway activation in various types of cancer.

In conclusion, we show herein that LXR agonists inhibited the growth of various GC cells. This inhibition correlated with the translocation of LXRβ from the cytoplasm to the nucleus. After the nuclear translocation of LXRβ, treatment with LXR agonists suppresses the activation of Wnt signalling and inhibits the proliferation of GC cells. This study suggests that LXRβ is a potential target in cancer therapy.

## DISCLOSURE STATEMENT

The authors declare that they have no conflicts of interest related to this work.
